# Inhibition of Quinolone- and Multi-Drug-Resistant Clostridioides Difficile Strains by Multi Strain Synbiotics—An Option for Diarrhea Management in Nursing Facilities

**DOI:** 10.3390/ijerph18115871

**Published:** 2021-05-30

**Authors:** Henning Sommermeyer, Hanna M. Pituch, Dorota Wultanska, Paulina Wojtyla-Buciora, Jacek Piatek, Malgorzata Bernatek

**Affiliations:** 1 Department of Health Sciences, Calisia University-Kalisz, Nowy Swiat 4, 62-800 Kalisz, Poland; h.sommermeyer@akademiakaliska.edu.pl (H.S.); paulinawojtyla@gmail.com (P.W.-B.); m.bernatek@akademiakaliska.edu.pl (M.B.); 2 Department of Medical Microbiology, Medical University of Warsaw, ul. Żwirki i Wigury 61, 02-091 Warsaw, Poland; hanna.pituch@wum.edu.pl (H.M.P.); dorota.wultanska@wum.edu.pl (D.W.)

**Keywords:** antibiotics, *Clostridioides difficile*, gut microbiota, multi-drug resistance, nursing facility, pathogen inhibition, prebiotics, probiotics, ribotype 027, synbiotics

## Abstract

Diarrhea is a common problem in nursing homes. A survey among nursing facilities in Poland was used to characterize diarrhea outbreaks, the burden caused for residents and caregivers and the employed measures. Survey results confirmed that diarrhea is a common problem in nursing homes and in most cases affects groups of residents. The related burden is high or very high for 27% of residents and 40% of caregivers. In 80% of nursing facilities pro or synbiotics are part of the measures used to manage diarrhea. Administration of these kinds of products has been suggested for the management of diarrhea, especially in cases caused by *Clostridioides (C.) difficile*. *C. difficile* is one of many potential causes for diarrhea, but is of particular concern for nursing homes because it is responsible for a large proportion of diarrhea outbreaks and is often caused by multi-drug resistant strains. In vitro inhibition of a quinolone-resistant and a multi-drug resistant *C. difficile* strain was used to evaluate the growth inhibitory effects of commonly used products containing probiotic microorganisms. Growth of both strains was best inhibited by multi-strain synbiotic preparations. These findings suggest that multi-strain synbiotics can be considered as an interventional option for diarrhea caused by *C. difficile*.

## 1. Introduction

Diarrhea is commonly accepted as a problem in nursing home facilities for the elderly, but few studies have evaluated its characteristics and how it is managed in daily practice. The present study employed a simple and easy to answer survey among heads of nursing staff of Polish nursing facilities for the elderly to collect information about: (i) occurrence rates and characteristics of diarrheal disease, (ii) the burden caused by diarrhea for residents and caregivers and (iii) the measures which are employed to manage diarrhea. The survey results indicated a surprisingly high (80%) utilization rate of probiotics or synbiotics for the management of diarrhea in nursing facilities. Based on this finding the study was extended to include an experimental part, aimed to characterize the effects of probiotics and synbiotics on the in vitro growth of *C. difficile*. *C. difficile* infection (CDI) is one of many possible reasons for diarrhea outbreaks in nursing home facilities for the elderly. However, in quantitative terms it is responsible for a large share of these outbreaks [1,2]. Infections with *C. difficile* are of great concern in nursing homes for the elderly for several reasons: (i) the spread of this spore-forming bacterium is difficult to control, (ii) the elderly residents of nursing homes are particular vulnerable to this type of infection which can have fatal consequences in severe cases and (iii) the growing spread of antibiotic-resistance among *C. difficile* strains, which is making management of these kind of infections with antibiotics challenging.

Multiple factors are contributing to the high incidence of CDI in nursing facility residents, among them are advanced age, close-proximity of residents, reduced diversity of the gut microbiota in the elderly, recent hospitalizations, prevalent comorbid illnesses and frequent exposure to antibiotics [3]. Antibiotics are among the most frequently prescribed medications in long term nursing facilities [4,5]. Some antibiotics, e.g., clindamycin, cephalosporins and fluoroquinolones, have been demonstrated to promote CDI [6,7,8]. The underlying mechanism of this side effect of antibiotic therapy is most likely a disruption of the normal gut microbiota, causing a loss of colonization resistance followed by an overgrowth of the gut by *C. difficile* [9,10,11]. This overgrowth can result in disease symptoms ranging from diarrhea to life-threatening clinical manifestations such as pseudomembranous colitis. 

During the last two decades the epidemiology of CDI has radically changed worldwide. The emergence of a hypervirulent epidemic *C. difficile* strain (PCR-ribotype 027/ North American pulsed-field electrophoresis type 1 (NAP1)/restriction endonuclease analysis (REA) BI type (BI)) shortly after the turn of the millennium is associated with an increased incidence and severity of disease [12]. *C. difficile* ribotype 027 strains are characterized by a hyper-production of TcdA and TcdB toxins which are the major virulence factors of *C. difficile* [13]. High prevalence rates of *C. difficile* ribotype 027 strains in nursing facilities were shown in a number of studies [14,15,16]. 

The usual treatment for primary and recurrent CDI requires the use of antibiotics with activities against *C. difficile*, and includes metronidazole, vancomycin, and fidaxomicin. The choice of antibiotic treatment is dependent on the severity of CDI as per the recommendations of the European Society of Clinical Microbiology and Infectious Diseases (ESCMID) [17] and the Infectious Diseases Society of America (IDSA) [18]. Development and spread of resistance against antibiotics have become an increasing problem for the treatment of CDI. Antimicrobial susceptibility studies of *C. difficile* revealed resistance to clindamycin, erythromycin, cephalosporins, and fluoroquinolones in high percentages of *C. difficile* clinical isolates [19]. Multi-drug resistance of *C. difficile*, defined as resistance to at least three classes of antimicrobial agents, has become a widespread problem and is now also commonly found in ribotype 027 strains [20,21,22]. While still effective for most cases of CDI, *C. difficile* isolates, among them those of the hypervirulent ribotype 027, have been shown to exhibit significantly reduced susceptibility to metronidazole [21,23] and vancomycin [21,23,24,25].

To limit further spread of antibiotic resistance in *C. difficile*, good antibiotic stewardship in all healthcare settings is of utmost importance. At the same time, alternative approaches for the management of CDI, for example the usage of preparations containing probiotic microorganisms (probiotics or synbiotics), receive growing interest as prophylactic measures, at least for patients at risk, (e.g., the elderly in nursing facilities), as complementary therapy during and after antibiotic therapy, or even as standalone therapy under certain specific circumstances for bacterial infections. 

In a number of studies evidence was shown that probiotics and/or synbiotics could play a beneficial role in the management of CDI. In vitro growth inhibition of *C. difficile* has been demonstrated for mono strain [26,27] and multi strain probiotics or synbiotics [28]. Administration of *Lactobacillus acidophilus* and *Bifidobacterium bifidum* seems to have a neutralizing effect on the toxins of *C. difficile*, as it was shown that only 46% of patients who received the probiotics were toxin-positive, compared to 78% of patients in the placebo group [29]. Colonization of *C. difficile* to epithelial cells could be prevented by administering a mixture of *Staphylococcus*, *Enterococcus*, *Lactobacillus*, *Anaerostipes*, *Bacteroidetes* and *Enterorhabdus* [30]. The yeast probiotic *Saccharomyces (Sac.) boulardii* upregulated the expression of anti-TcdA secretory immunoglobulin A in animal models of CDI and inhibited the binding of TcdA to epithelial cells [31,32]. A mixed culture of non-toxigenic *C. difficile*, *Escherichia coli*, *Bifidobacterium bifidum* and members of *Lachnospiraceae* was shown to prevent the colonization of *C. difficile* in germ-free mice [33,34]. In a meta-analysis study [35] it has been shown that probiotics are associated with a reduction in the incidence of CDI-associated diarrhea. A review published in 2008 by the Cochrane Group [36] stated that there was not enough data to establish the role of probiotics for the treatment of CDI. In a more recent systematic review and meta-analysis by the Cochrane Collaboration, published in 2017, the authors came to the conclusion that probiotics have general positive effects in CDI patients [37]. Currently there is still not sufficient data to support a positive recommendation to use products containing probiotic microorganisms for the management of CDI. Nevertheless, these products are known to be used in healthcare, however, to what extent they are used is not well characterized. 

In the experimental part of the present study the effects of some commonly used probiotic and synbiotic preparations on the in vitro growth of *C. difficile* were characterized. Two different *C. difficile* strains were employed in the experiments to address the recent dynamics in the evolution of antibiotic resistance in *C. difficile*. One of the investigated strains, *C. difficile* (ATCC^®^ 9689™), is a well characterized strain of the ribotype 001, which is commonly used as a reference strain in microbiological laboratories. This strain is resistant against inhibition by fluoroquinolones, but is still susceptible against most other antibiotics. Antibiotic susceptibility testing (AST) was performed to confirm the resistance profile of this *C. difficile* strain. The second strain, *C. difficile* No. 644, was originally isolated from a symptomatic CDI patient in the course of a surveillance study performed in Polish hospitals in 2012 [38]. *C. difficile* No. 644 belongs to the group of hypervirulent epidemic ribotype 027 *C. difficile* strains, which are today commonly observed in nursing facilities for the elderly [14,15,16]. AST of this strain was performed to establish its antibiotic resistance profile.

The results of the head-to-head in vitro growth inhibition by different products will allow healthcare professionals to make a more educated decision when selecting from the numerous available probiotic and synbiotic products for the management of their CDI patients.

## 2. Materials and Methods

### 2.1. Surveys

A cross-sectional study, which included a survey among heads of nursing staff of Polish nursing facilities for the elderly, was performed by sending a short cover letter outlining the objectives of the research project and a one-page questionnaire comprising eight questions (Table 1) by regular post.

Postal addresses of the nursing facilities were taken from the webpages of the nursing homes. The heads of nursing staff were provided with an e-mail address to which they were invited to send the completed questionnaire. A total of 200 nursing facilities in Poland were contacted. One reminder was sent to nursing homes who had not responded within two weeks of the first contact. Data processing was approved by responders by stamp, date and their signature. No incentive of any kind was provided to responders. However, responders could mark a box indicating that they were interested to be informed about the results of the survey. Answers from returned questionnaires were collected in a database created with the software Excel (Microsoft, Redmond, WA, USA). Free-text answers were documented in the same database as full text. Keywords in the free-text answers were identified and used as basis to analyze this type of answer.

### 2.2. Analyses of Questionnaires

Questionnaires were collected and the changes of the percent values of responders of any of the possible pre-defined answers by the last ten collected questionnaires were analyzed. Questionnaires were collected until the answers of the last ten received questionnaires were not changing the percent value of any of the pre-defined answers by more than 5%. Changes caused by the last ten questionnaires received were on average 0.4%, with −2.1% being the largest negative and +3.6% being the largest positive change. Some of the questions were left with no answers. Such data points were treated as missing values.

### 2.3. Probiotics and Synbiotics

The yeast *Sac. boulardii* probiotic Enterol^®^ (Biocodex, Gentilly, France) contains in each capsule 4.5 × 10^9^ colony forming units (CFU) of the *Sac. boulardii* strain CNCM I-745. Dicoflor^®^ (Bayer Sp. z o.o., Warszawa, Poland) contains 6 × 10^9^ CFU of *Lacticaseibacillus rhamnosus GG* ATCC^©^ 53103 per capsule. BioGaia^®^, (InfectoPharm Arzneimittel und Consilium GmbH, Heppenheim, Germany) contains 10^8^ CFU *Limosilactobacillus reuteri DSM 17938* per 5 drops. Lakcid^®^ (Biomed-Lublin S.A., Lublin, Poland) contains a total of 2 × 10^9^ CFUs as mixture of the *Lacticaseibacillus rhamnosus* strains *E/N* (40%), *Oxy* (20%) and *Pen* (40%), [39]. The multi strain synbiotic A (Multilac^®^ Synbiotic, Vivatrex GmbH, Aachen, Germany) contains in each capsule 9.00 × 10^8^ *Lactococcus lactis Ll-23*, 9.00 × 10^8^ CFUs *Lactobacillus helveticus SP 27*, 6.75 × 10^8^ CFUs *Bifidobacterium longum Bl-05*, 4.5 × 10^8^ CFUs *Bifidobacterium breve Bb-03*, 4.5 × 10^8^ CFUs *Lacticaseibacillus rhamnosus Lr-32*, 4.5 × 10^8^ CFUs *Streptococcus thermophiles St-21*, 2.25 × 10^8^ CFUs *Lacticaseibacillus casei Lc-11*, 2.25 × 10^8^ CFUs *Lactiplantibacillus plantarum Lp-115*, 2.25 × 10^8^ CFUs *Bifidobacterium bifidum Bb-02*, and 68 mg of the prebiotic fructooligosaccharides (FOS). The multi strain synbiotic B (Multilac^®^ Baby, Vivatrex GmbH, Aachen, Germany) is a freeze-dried powder. Each sachet contains a total of 10^9^ CFUs as a mixture of equal CFU amounts of *Lactobacillus acidophilus LA-14*, *Lacticaseibacillus casei R0215*; *Lacticaseibacillus paracasei Lpc-3*; *Lactiplantibacillus plantarum Lp-115*; *Lacticaseibacillus rhamnosus GG*, *Ligilactobacillus salivarius Ls-33*, *Bifidobacterium lactis Bl-04*, *Bifidobacterium bifidum R0071*, *Bifidobacterium longum R0175* and 1.43 g of FOS.

### 2.4. C. difficile Strains

The ribotype 001 strain *C. difficile* (ATCC^®^ 9689™) was purchased from ATCC, Manassas, Virginia, USA [40].

The *C. difficile* No. 644 strain is member of a collection of *C. difficile* strains that has been established in the course of a surveillance study conducted in 2012 to obtain an overview of CDI in Polish hospitals [38]. Ethical approval and informed consent were not required. The strain was isolated from a symptomatic CDI patient, diagnosed on the basis of the CDI definitions of 2012 proposed by ESCMID [41]. For the isolation of the strain, the fecal sample was inoculated anaerobically on selective media for 48 h, and *C. difficile* colonies were sub-cultured on blood-agar and identified using standard methods, as described previously [42]. PCR-ribotyping of the isolate was performed by the Anaerobe Laboratory, Medical University of Warsaw according to the method described by Stubbs et al. [43]. The Cardiff-ECDC collection of reference isolates (n = 23) of *C. difficile* was used as reference set.

### 2.5. Antimicrobial Susceptibility Testing (AST)

AST of the C. difficile strains was performed by using the gradient diffusion method ETEST^®^ for epidemiological research (bioMérieux SA, Marcy l’Etoile, France). Tests with ETEST^®^ strips that contained gradients of each agent tested were performed as specified by the producer [44]. The following antimicrobials were tested: metronidazole (MZ) and vancomycin (VA), clindamycin (CLI), erythromycin (ERY), which had ETEST^®^ strips ranging from 0.016 to 256 mg/L; and ciprofloxacin (CIP), moxifloxacin (MXF), imipenem (IP), which had ETEST^®^ strips ranging from 0.002 to 32 mg/L. Minimum inhibitory concentration (MIC) values were read from the scales in terms of mg/L at complete inhibition of growth of the respective C. difficile strain. European Committee on Antimicrobial Susceptibility Testing (EUCAST) clinical breakpoints for C. difficile were applied to the antimicrobial drugs MXF, MZ and VA (EUCAST. Available online: www.eucast.org (assessed on 29 May 2021)) [45]. For CIP, CLI, ERY and IP, Clinical and Laboratory Standards Institute (CLSI) clinical breakpoints were assessed [46].

### 2.6. In Vitro Growth Inhibition of C. difficile Strains

For the in vitro pathogen inhibition studies with the different *C. difficile* strains, the pathogens were cultivated under anaerobic conditions at 35–37 °C for 24–48 h on Schaedler agar (CM0437, Fisher Scientific GmbH, Schwerte, Germany) [47]. Suspensions of the evaluated products each containing 10^6^ CFU were inoculated on MRS agar and incubated for 48 h in the presence of 5% CO_2_. 10 mm diameter bars were transferred to a Mueller–Hinton agar with 5% horse blood and 20 mg/L NAD (PP0972, E&O Laboratories Ltd., Bonnybridge, UK) and incubated under anaerobic conditions for 24 h.

### 2.7. FOS Control and Measurement of Growth Inhibition

For testing a potential pathogen growth inhibitory effect of FOS, 100 μL of a solution containing 14.3 mg/mL FOS (F8052, Sigma Aldrich, St. Louis, MI, USA) was applied to a 10 mm filter disk that was then administered to respective pathogen testing plates. The multi strain synbiotics A and B containing nine different probiotic strains were tested on the same plates as positive controls.

At the end of the incubation, measurements of inhibition zones around the tested colonies were taken from the outer edge of the colonies to the outer edge of the clear zones. Each test was performed in triplicate and the arithmetic means of the radii measuring from the edges of the colonies to the edges of the clear zones were calculated, as well as the standard deviations SD (Excel, Microsoft, Redmont, Washington, WA, USA). Independent T-test statistical analyses of datasets were conducted with GraphPad Prism software version 8.2 (GraphPad Software, San Diego, CA, USA). Datasets were considered as significantly different when a *p*-value < 0.01 was achieved.

## 3. Results

### 3.1. Survey Results

From July to October 2020, responses from 59 nursing facilities for the elderly (responder rate 29.5%) in Poland were obtained. Characteristics of the responding nursing facilities in terms of size (number of resident places) and age structure of the residents are shown in Table 2.

Among the answered questionnaires were four with one non-answered questions. One of the surveys did not specify the average age of the residents (question 2) and three missed the estimate for the number of residents affected by diarrhea at least once during a year (question 3).

**Figure 1:**
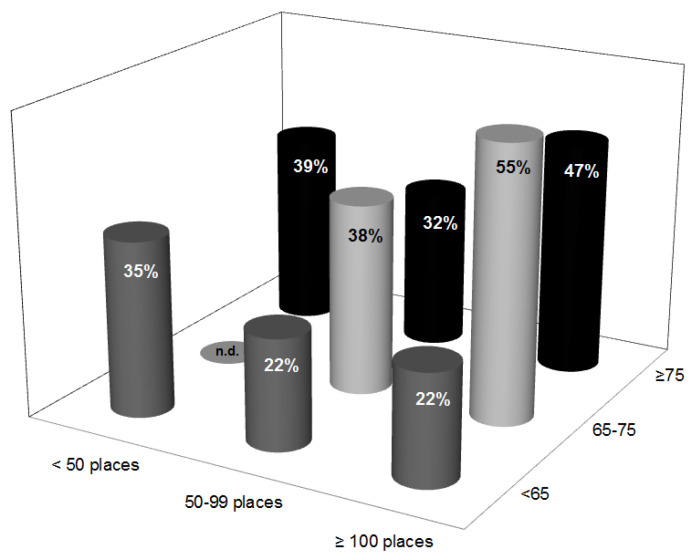
Average percentage of residents, indicated by column height, who had diarrhea at least once a year as function of nursing home size and average age of residents (total number of answers n = 55). Values were only calculated in cases where at least three answers were available for the respective size/age-category (n.d. = insufficient number of data).

The average number of residents affected at least once during the last year by diarrhea varied largely. However, diarrhea is affecting on average at least about one fifth of all residents in nursing homes in a given year. Based on the collected data, diarrhea seems to be a bigger issue in larger nursing homes with older residents (Figure 1).

**Figure 2:**
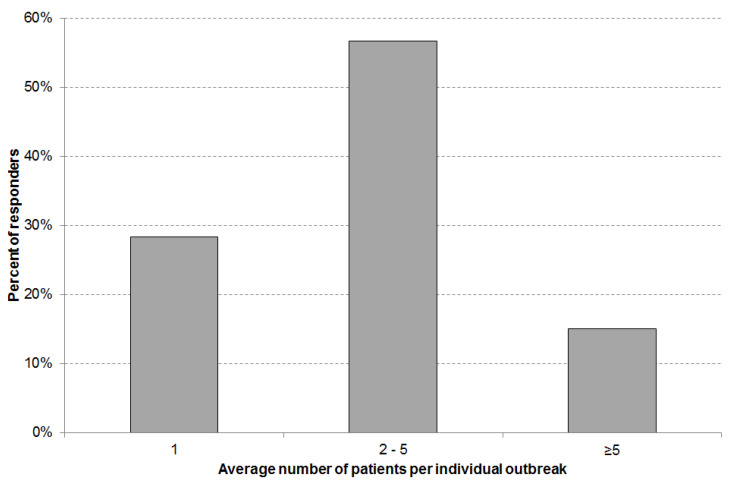
Average number of patients affected by an individual diarrhea outbreak (total number of answers n = 59).

In the majority of cases (72%) diarrhea outbreaks affected more than one resident (Figure 2). In 15% of cases the diarrhea spread to five or more residents.

**Figure 3:**
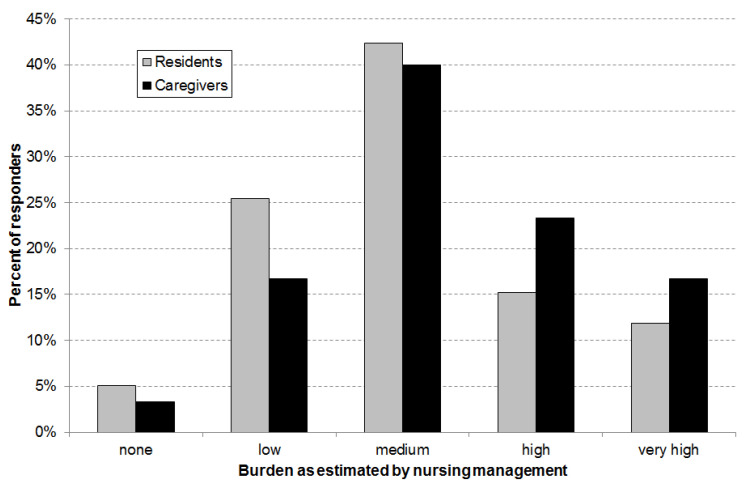
Burden related to diarrhea for nursing home residents and care givers as estimated by the nursing home management (total number of answers for burden for residents of n = 59 and for burden for care givers n = 59).

The burden caused by diarrhea was assumed to be high or very high for 27% of the residents and 40% of the caregivers (Figure 3). Overall, the burden for caregivers was rated higher than that of the residents.

**Figure 4:**
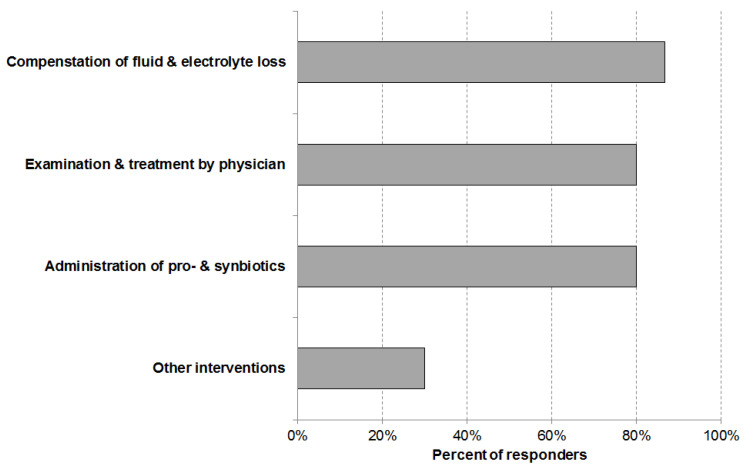
Interventions employed for the management of diarrhea cases in nursing facilities (total number of answers n = 59).

Common measures for the management of diarrhea (Figure 4) are (i) “compensation of fluid and electrolyte loss” (87%), (ii) “examination and treatment by a physician” (80%), and “administration of pro or synbiotics” (80%). Other interventions were mentioned by 30% of the responders. In most of the cases the “other intervention” was a change in diet.

### 3.2. Antimicrobial Susceptibility of the C. difficile Strains

Results from AST of the two *C. difficile* strains are provided in Table 3. *C. difficile* (ATCC^®^ 9689™) is resistant against the quinolones ciprofloxacin and moxifloxacin. *C. difficile* No. 644 exhibits a multi-drug resistance profile with resistances against quinolones, clindamycin, erythromycin, and imipenem. Both strains are susceptible for inhibition by metronidazole and vancomycin.

### 3.3. In Vitro Inhibition of the C. difficile Strains by Different Products Containing Probiotic Microorganisms

**Figure 5:**
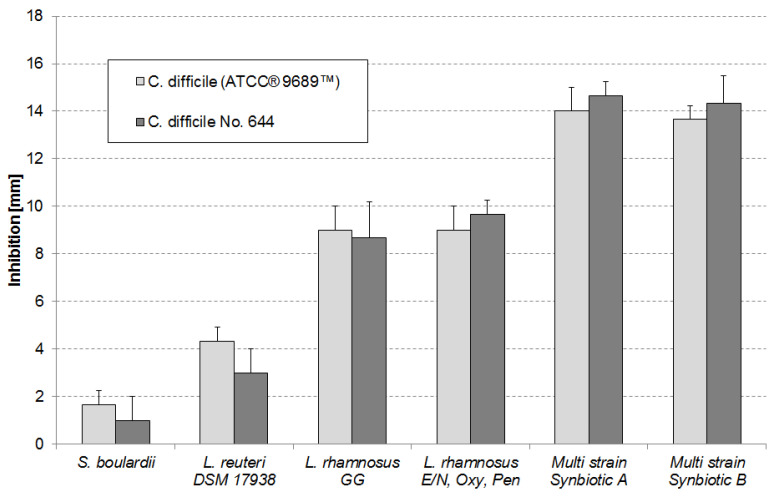
In vitro growth inhibition of *C. difficile* strains by different probiotics and two multi strain synbiotics. The *L. rhamnosus E/N*, *Oxy*, *Pen* mixture contains the three different probiotics in a CFU ratio of 40%/20%/40%. Detailed information about the composition of the multi strain synbiotics (containing 9 different bacterial probiotic and FOS) are provided under Materials and Methods.

No significant differences (*p*-value > 0.05) were observed between the inhibition of the two *C. difficile* strains by each of the tested products containing probiotic microorganisms (Figure 5). In contrast, inhibition of the strains by the individual products differed largely. Smallest inhibition was observed by the yeast *Sac. boulardii*. Slightly stronger inhibition was shown for the mono strain probiotic containing L. reuteri DSM 17938. Inhibition by the mono strain *L. rhamnosus GG* probiotic and the multi strain probiotic containing the three *L. rhamnosus* strains E/N, Oxy and Pen were very similar (*p*-value = 0.5) for the *C. difficile* (ATCC^®^ 9689™) strain and for the *C. difficile* No. 644 strain (*p*-value = 0.17). Strongest inhibitions of both strains were observed for the multi strain synbiotics containing nine different probiotic bacteria and FOS. There was no significant difference between the inhibitions of each *C. difficile* strain by the two multi strain products (*p*-value = 0.19 for the multi strain synbiotic A and *p*-value = 0.21 for the multi strain synbiotic B). Inhibitions by the multi strain synbiotics were significantly stronger (*p*-values < 0.05) compared to all other tested products. FOS alone caused no inhibition of the *C. difficile* strains (data not shown).

## 4. Discussion

In 2019, 81,800 residents were living in 875 nursing facilities for the elderly in Poland [48]. The present study contacted nearly a quarter of these facilities and obtained answers from seven percent (responder rate 30%). Results of the survey confirmed that diarrhea is a frequent problem in nursing facilities for the elderly. At least every fifth resident of a nursing facility will experience at least one diarrhea event in a given year. For residents of nursing facilities with an older resident population and for those living in facilities with a larger number of residents, the likelihood of encountering diarrhea seems to be even higher. The latter resonates with older age and closer proximity to a larger number of other residents being among the risk factors for the occurrence of diarrhea, which is most frequently caused by CDI in nursing facilities for the elderly [49,50,51]. In most cases (>70%) diarrhea will not stay with one individual resident but will spread to other residents. In about 15% of cases, diarrhea outbreaks will affect five or more residents. Diarrhea causes a “high” or “very high” burden for the residents (27%) and the caregivers (40%), indicating that avoiding diarrhea in nursing facilities will relieve stress for residents and even more for their caregivers. The most common management of diarrhea in nursing facilities involves the compensation of fluid and electrolyte loss (87%), which in most cases (80%) involves a physician performing patient examinations and making treatment decisions. Interestingly enough, in 80% of the responding nursing facilities in Poland, the administration of pro or synbiotics is part of the established routine for the management of diarrhea. This rate is surprisingly high, as study data showing beneficial effects of these products are still limited and they are not part of the treatment guidelines for diarrhea management [Goldenberg et al. 2017]. Other interventions mentioned for the treatment of diarrhea in nursing facilities were changes of the diet of residents, indicating that diet is frequently considered as a source of the problem.

In the interest of a high responder rate, the questionnaire employed for the present study was kept simple and easy to answer. This approach limited the possibility for an in-depth evaluation of the topic which had to be reserved for a later study employing a larger and more comprehensive set of questions. Assessing the problem of diarrhea in nursing home facilities by questioning the nursing facilities’ staff has some general limitations. Answers might be biased by the responders’ intention to down-play the problem as it might harm the reputation of the nursing facility and its staff. Answers regarding the burden for residents also should be interpreted with care, as the residents’ burden caused by diarrhea might be very subjectively perceived by their caregivers. At the same time, it has to be realized that asking the residents of nursing homes themselves would cause numerous (methodological and legal) problems. Despite these limitations, based on the achieved consistency of the obtained answers, there is reason to believe that the survey provides a reasonably accurate assessment of problems relating to diarrhea in the nursing facility setting.

While diarrhea outbreaks in nursing facilities for the elderly can have a number of causes, they are frequently related to CDI infections spreading among residents. In this regard it is a reasonable approach of nursing homes to employ probiotics and synbiotics for the management of diarrhea outbreaks. Probiotics and synbiotics are claimed to support the colonization resistance of the gut microbiota which is discussed as an essential mechanism of limiting the spread of *C. difficile* and of other bacterial pathogens. With the lack of recommendations and guidelines for the use of products containing probiotic microorganisms for the management of CDI-related diarrhea in nursing homes [17,18], product selection by the nursing home staff is often not supported by clinical or non-clinical data. The results from the in vitro experiments of the present study, comparing some commonly used products containing probiotic microorganisms, can provide at least some orientation. For the in vitro experiments two *C. difficile* strains were selected. The *C. difficile* (ATCC^®^ 9689™) strain is used as a reference in microbiology laboratories around the world. The *C. difficile* No. 644 strain represents the category of hypervirulent epidemic ribotype 027 *C. difficile*, which is highly relevant for diarrhea in nursing homes. Antimicrobial susceptibility testing revealed that the *C. difficile* (ATCC^®^ 9689™) strain was resistant to quinolones (ciprofloxacin and moxifloxacin). The ribotype 027 *C. difficile* strain No. 644 exhibited multi-drug resistance, defined as resistance against at least three classes of antimicrobial agents. The strain was shown to be resistant against ciprofloxacin, moxifloxacin, clindamycin, erythromycin and imipenem. Whenever nursing facility residents are treated with antibiotics, physicians have to take into consideration that the resident might be a carrier, with or without symptoms, of a *C. difficile* strain which might be multi-drug resistant and therefore might not be sensitive to the administered antibiotic. In these cases, the administered antibiotic will provide a growth advantage to *C. difficile* compared to other antibiotic-susceptible bacteria in the gut. This can result in overgrowth in the gut of *C. difficile* and CDI disease manifestation.

A diverse and balanced gut microbiota is assumed to be the best protection against the overgrowth in the gut of *C. difficile* [9,10,11], therefore, supplementing the gut by administration of products containing probiotic microorganisms is a sensible approach. There is a great variety of these kinds of products available, which can be differentiated by the type of probiotic microorganism (yeast or bacteria), number of probiotic strains (mono strain or multi strain) and the presence or absence of a prebiotic component (probiotics or synbiotics). In the present study, in vitro inhibitory effects of a number of commonly used products on the growth of *C. difficile* were evaluated as these can be considered as surrogate measures for the effectiveness in CDI patients or in *C. difficile* carriers. The results demonstrated that the inhibitions by products were similar for both tested *C. difficile* strains and not a function of the strains’ antibiotic susceptibility. A similar finding has recently been demonstrated for different strains of *Klebsiella pneumoniae* [52]. In vitro growth inhibitions of the *C. difficile* strains varied largely among the individual products, with the tested multi strain synbiotics showing the strongest inhibitions. A potential reason for the superior effects of the multi strain synbiotics might be synergistic effects among the different probiotic bacteria, leading to a stronger overall inhibitory effect on the growth of *C. difficile* [53,54,55].

## 5. Conclusions

The spread of hypervirulent epidemic *C. difficile* strains, (e.g., ribotype 027), often being multi-drug resistant against major categories of antibiotics, has become a growing concern for healthcare worldwide. The possibility to inhibit the in vitro growth of a multi-drug resistant *C. difficile* strain by certain multi strain synbiotics suggests that these products can play a beneficial role in the management of CDI. More clinical studies are needed to further characterize the potential role of multi strain synbiotics in clinical practice.

## Figures and Tables

**Table 1:** Questions in the survey.

No	Question	Type of Answer
1	To which size category would you assign your nursing home?	Selection of one of the pre-defined answers
2	What is the average age of your residents?	Selection of one of the pre-defined answers
3	What percentage of your residents have diarrhea at least once a year?	Percentage number
4	In case diarrheal diseases occur, how many residents are usually affected?	Selection of one of the pre-defined answers
5	How would you rate the burden on residents caused by diarrhea?	Selection of one of the pre-defined answers
6	How would you rate the burden on caregivers caused by diarrhea?	Selection of one of the pre-defined answers
7	What measures are employed to manage diarrhea cases?	Multiple selection of predefined answers and field for free-text answer
8	Are you interested in diarrheal diseases in elderly and nursing homes?	Selection of one of the pre-defined answers

**Table 2:** Characteristics of responding nursing facilities.

		Size of Nursing Facilities			
		<50 Places	50–99 Places ^1^	≥100 Places	All
Average Age of Residents	<65	3	9	7	19
	65–75	1	7	9	17
	≥75	6	6	10	22
	all	10	22	26	58

^1^ One nursing home of this size category did not provide information about the average age of its residents and therefore was eliminated from this overview.

**Table 3:** Antimicrobial susceptibility of *C. difficile* strains determined by using the gradient diffusion method ETEST^®^.

Antibiotic	*C. difficile* (ATCC^®^ 9689™) MIC ^1^ [mg/L]	*C. difficile* No. 644 MIC ^1^ [mg/L]	Resistance Breakpoint [mg/L]
Ciprofloxacin	32	32	>4 (CLSI ^2^)
Moxifloxacin	32	32	>4 (EUCAST ^3^ v.11.0, ECOFF ^4^)
Clindamycin	3	32	>8 (CLSI)
Erythromycin	0.38	256	>8 (CLSI)
Imipenem	8	32	>8 (CLSI)
Metronidazole	0.5	0.38	>2 (EUCAST v. 11.0)
Vancomycin	1.0	0.5	>2 (EUCAST v. 11.0)

^1^ Minimum inhibitory concentration. ^2^ Clinical and Laboratory Standards Institute. ^3^ European Committee on Antimicrobial Susceptibility Testing ^4^ Epidemiological cut off.

## Data Availability

The data presented in this study are openly available in FigShare at https://doi.org/10.6084/m9.figshare.14701275.v1 (accessed on 29 May 2021).
